# Ripping the Ripoptosome: a novel path for blocking allergic inflammation?

**DOI:** 10.1038/s41423-021-00815-4

**Published:** 2022-01-07

**Authors:** Theresa Neuper, Richard Weiss, Jutta Horejs-Hoeck

**Affiliations:** University of Salzburg, Department of Biosciences, Hellbrunner Str. 34, 5020 Salzburg, Austria

**Keywords:** Innate immunity, Signal transduction

Scientists at Cincinnati Children’s Hospital Medical Center report that an innate molecular signaling hub known as the ripoptosome plays a key role in the development of allergic pathologies and suggest that manipulation of this response platform could open new paths for the treatment of allergic diseases [1].

Allergies are an unremitting global health care problem. Although the symptoms of affected individuals are well documented for a wide range of allergic disorders, the precise molecular mechanisms that trigger allergic inflammation to innocuous substances remain elusive. Thus, a key question in allergy research is why certain environmental proteins can trigger the induction of allergic Th2 responses. Seminal work by Ruslan Medzhitov’s group identified protease activity inherent in many allergens as being critical to eliciting the hypersensitivity response. Although their earlier work indicated basophils as important protease sensors for promoting type 1 allergies [2], it soon emerged that intraepithelial T cells also play a role by linking lymphoid stress surveillance in epithelia to atopy [3] with IL-33 representing a central player in this signaling cascade. The alarmin IL-33 is a member of the IL-1 cytokine family, and its critical role in the context of allergic inflammation is well established. Upon secretion into the extracellular space, IL-33 binds to ILC2s, mast cells, Th2 cells, eosinophils, basophils and dendritic cells, all of which stably express ST2, which, along with its coreceptor, the IL-1 receptor accessory protein (IL-1 RAcP), forms the heterodimeric receptor for IL-33 [4]. Triggering the IL-33/ST2 axis activates and recruits various Th2-related cell types, thereby contributing to the development and amplification of an allergic inflammatory response. However, detailed information on the molecular mechanisms of how allergens trigger IL-33 production in epithelial cells has remained scarce.

In a masterful paper published in *Nature Immunology* in October 2021, Brusilovsky and colleagues established a new milestone by identifying the ripoptosome as a novel signaling hub involved in the innate response to various allergens [1]. The authors elegantly describe how epithelial cells contribute to the onset of allergic airway inflammation by activating the ripoptosome, which subsequently drives the maturation and secretion of IL-33. First, the authors showed that allergens derived from a wide range of organisms, including fungi, cockroaches and mites, but not pollen or food allergens, induce intracellular maturation and subsequent secretion of IL-33 by epithelial cells. This was a completely unexpected finding because maturation of IL-33 induced by allergens was believed to be an exclusively extracellular event mediated by allergen proteases [5]. By employing a pan-caspase inhibitor to block intracellular caspase activity, Brusilovsky et al. showed that allergen-induced caspase 8 activation is required for the cleavage of downstream caspases 3 and 7, which ultimately results in the intracellular processing of IL-33. Experiments using epithelial cells overexpressing the intracellular IL-33 precursor revealed that caspase 3 and caspase 7, but not caspase 8, cleave premature IL-33 into the biologically active mature IL-33 protein. Specific inhibition of caspase 8, however, completely abolished the release of mature IL-33, confirming that activation of caspase 8 is a prerequisite for the intracellular cleavage of IL-33 by downstream caspases 3 and 7.

**Fig. 1:**
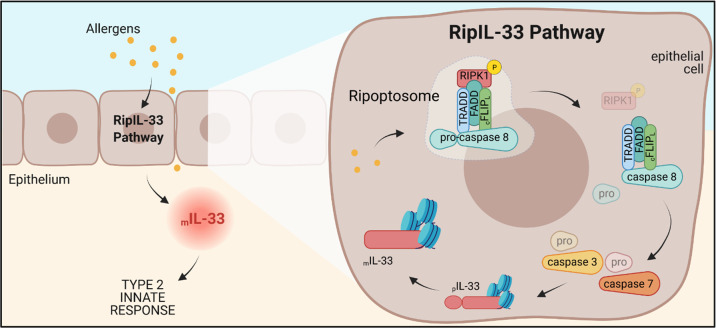
Activation of the RipIL-33 pathway drives the onset of type 2 innate responses. Brusilovsky and colleagues describe how various allergenic stimuli converge in the activation of the ripoptosome and how this contributes to the onset of type 2 innate responses. The authors provide evidence that allergen recognition by epithelial cells results in the activation of the RIPK-1/caspase 8 multiprotein complex called RipIL-33. Caspase 8 plays a key role in the RipIL-33 pathway. On the one hand, RIPK-1 is rapidly phosphorylated and subsequently degraded, thus attenuating death-inducing signals. On the other hand, caspase 8 promotes the maturation of IL-33 through targeted activation of caspase 3 and caspase 7 and corelease of histones. Figure created with BioRender.com

Intriguingly, allergen-dependent caspase 8 activation seems to require the ripoptosome, a “signaling platform” originally associated with cell death-inducing signals. RIPK-1, a key mediator of this complex, is rapidly phosphorylated upon allergen exposure, thereby activating caspase 8, which appears to mediate a functional shift of the ripoptosome from a ‘cell death’ state to an ‘allergy’ state by promoting rapid degradation of RIPK-1. This finding suggests that allergen-induced release of IL-33 by epithelial cells requires not only RIPK-1 phosphorylation but also subsequent degradation of phosphorylated RIPK-1, activation of caspase 8, and cleavage of downstream caspases 3 and 7. To emphasize this novel and unexpected link between ripoptosome activation and intracellular IL-33 processing, the researchers named this newly discovered pathway “RipIL-33” (Fig. 1).

To demonstrate that the IL-33 that is processed via the RipIL-33 pathway is biologically active and binds its cognate receptor ST2, Brusilovsky and colleagues applied *in silico* and in vitro experimental approaches. A minimal IL-33 binding domain interacting with ST2 was resolved by X-ray and Nuclear magnetic resonance (NMR) analyses, and the physical interaction between endogenous IL-33 and ST2 was confirmed by coimmunoprecipitation experiments. Using cell lines that express and secrete either premature IL-33 or mature isoforms of IL-33, the authors showed that mature IL-33 can bind and activate ST2. Prompted by the observation that mature IL-33 contains an intact chromatin-binding domain and is coreleased with histones, the authors further assessed the biological activity of mature IL-33 in the presence or absence of purified histones. These results demonstrated that, in addition to IL-33’s proteolytic maturation, histones, which are secreted upon ripoptosome activation, further potentiate the bioactivity of mature IL-33. To demonstrate the in vivo relevance of the RipIL-33 pathway in the context of an allergic response, the authors established a murine model of allergic airway inflammation induced by the fungal allergen *Alternaria alternata*. Genetic deletion and pharmacological inhibition of caspase 8 produced a significant decrease in lung inflammation upon allergen contact, which manifested as reduced leukocyte influx and diminished Th2-associated cytokine release into the lungs of challenged mice. Similarly, reduced eosinophil and neutrophil infiltration as well as the levels of IL-4, IL-5 and IL-13, already evident 4 h after *A. alternata* challenge on three consecutive days, conclusively demonstrated that allergen-induced innate type 2 responses are strongly RipIL-33 dependent. Future in vivo studies could aim to evaluate the contribution of RipIL-33 signaling to adaptive type 2 immune responses. Nonetheless, the authors neatly corroborated the clinical significance of the ripoptosome in the context of allergic inflammation by examining biopsies from patients with eosinophilic esophagitis, showing that ripoptosome activation markers and mature IL-33 levels dynamically correlated with the degree of esophageal eosinophilia and disease activity.

Intriguingly, this study describes a common biological response platform, initially known to induce cell death, as performing double duty as a novel allergen-sensing pathway, thus providing a ‘missing link’ in understanding the onset of allergic reactions. However, it remains unclear whether the allergen itself or specific components of the allergen context activate the RipIL-33 pathway. Although allergens can have enzymatic activity and thus pose a threat to the host, most allergens themselves do not promote type 2 inflammation per se [6, 7]. Thus, studying the allergen context will help to clarify whether allergens themselves or specific allergen-associated components are sensed by the ripoptosome. While the authors convincingly demonstrate that RipIL-33 activation contributes to innate immune responses related to type 2 inflammation, the consequences of caspase 8/ripoptosome activation on adaptive immunity and the relevance to other allergic conditions in humans, such as lung inflammation and asthma, still need to be investigated. Nevertheless, targeting the RipIL-33 pathway could provide a unique strategy to counteract type 2 immunity and alleviate allergic inflammation. Thus, the identification of drugs that specifically control the RipIL-33 response could benefit people suffering from a wide range of allergies.
